# Anesthesia management in a patient with anomalous origin of left pulmonary artery from the descending aorta: A case report and literature review

**DOI:** 10.3389/fsurg.2023.1140212

**Published:** 2023-03-15

**Authors:** Lu Wang, Yaru Li, Xinrui Yin, Qiaoyu Han, Yi Feng, Yi Shi, Gang Liu, Liang Sun

**Affiliations:** ^1^ Department of Anesthesiology, Peking University People’s Hospital, Beijing, China; ^2^ Department of Cardiac Surgery, Peking University People’s Hospital, Beijing, China

**Keywords:** adolescent, anomalous origin of the left pulmonary artery, surgical repair, pulmonary arterial hypertension, anesthesia management

## Abstract

Anomalous origin of the left pulmonary artery from the descending aorta is an extremely rare congenital malformation. There were merely four case reports of such malformation in previous literature, and all four cases underwent surgical repair in their first year of life. Actually, long-term pulmonary arterial hypertension and irreversible pulmonary vascular changes make anesthesia management quite a challenge, while anesthesia for managing these cases has not been discussed before. We present a 15-year-old boy undergoing corrective surgery and try to provide some tips on anesthesia management for this surgical procedure. Through optimal perioperative management, successful outcomes can be achieved for this malformation.

## Introduction

Anomalous origin of single pulmonary artery branch from aorta is a rare anomaly, which accounts for 0.12% of congenital heart disease (1). In clinical scenarios, anomalous origin of the right pulmonary artery represents the majority of cases, making anomalous origin of the left pulmonary artery (AOLPA) even much rarer (2). For AOLPA, the left lung is perfused under systemic pressure and the right lung accepts the whole output of right ventricle. Therefore, patients with AOLPA from the aorta are usually associated with higher mortality, and most survival corrections are achieved within the first month of age (3). To the best of our knowledge, there have been four case reports of AOLPA from the descending aorta of those who underwent corrective surgery within the first year of life in the literature (Table 1) (4, 5). While these reports mostly focused on the surgical operations, discussions on anesthesia for these cases are lacking. Herein, we present an adolescent undergoing the correction for AOLPA from the descending aorta as the fifth case and try to provide some tips on anesthesia management.

**Table 1:** The demographic and clinical characteristics in five patients with AOLPA from the descending aorta.

Patient	1	2	3	4	5
First author (reference)	Prifti (3)	Peng (4)	Gnanappa (1)	Rajanbabu (5)	The current case
Year	2003	2004	2016	2019	2023
Demographic data					
Age (m)	1.7	0.75	0.5	8	180
Sex	Male	Male	Male	Female	Male
Weight (kg)	4.6	NA	2.7	NA	90
Clinical data					
Syndromes	Waardenburg	Growth retardation	Increased work of breathing, poor feeding, desaturation	Recurrent respiratory tract infections	Aggravated hemoptysis
Associated anomaly	No	No	Patent ductus arteriosus	Patent ductus arteriosus	No
PAH	Mild	Moderate	NA	NA	Mild
Surgical data					
Surgical procedure	Graft	Implantation *via* synthetic vessel	DR	DR	Implantation *via* synthetic graft
CPB	No	No	Yes	No	Yes
Postoperative data					
Length of stay (days)	23	14	NA	5	27
Outcome	Survived	Survived	Survived	Survived	Survived
Follow-up (months)	68	56	NA	6	NA

CPB, cardiopulmonary bypass; DR, direct implantation; NA, not available; PAH, pulmonary arterial hypertension.

## Case description

**Figure 1:**
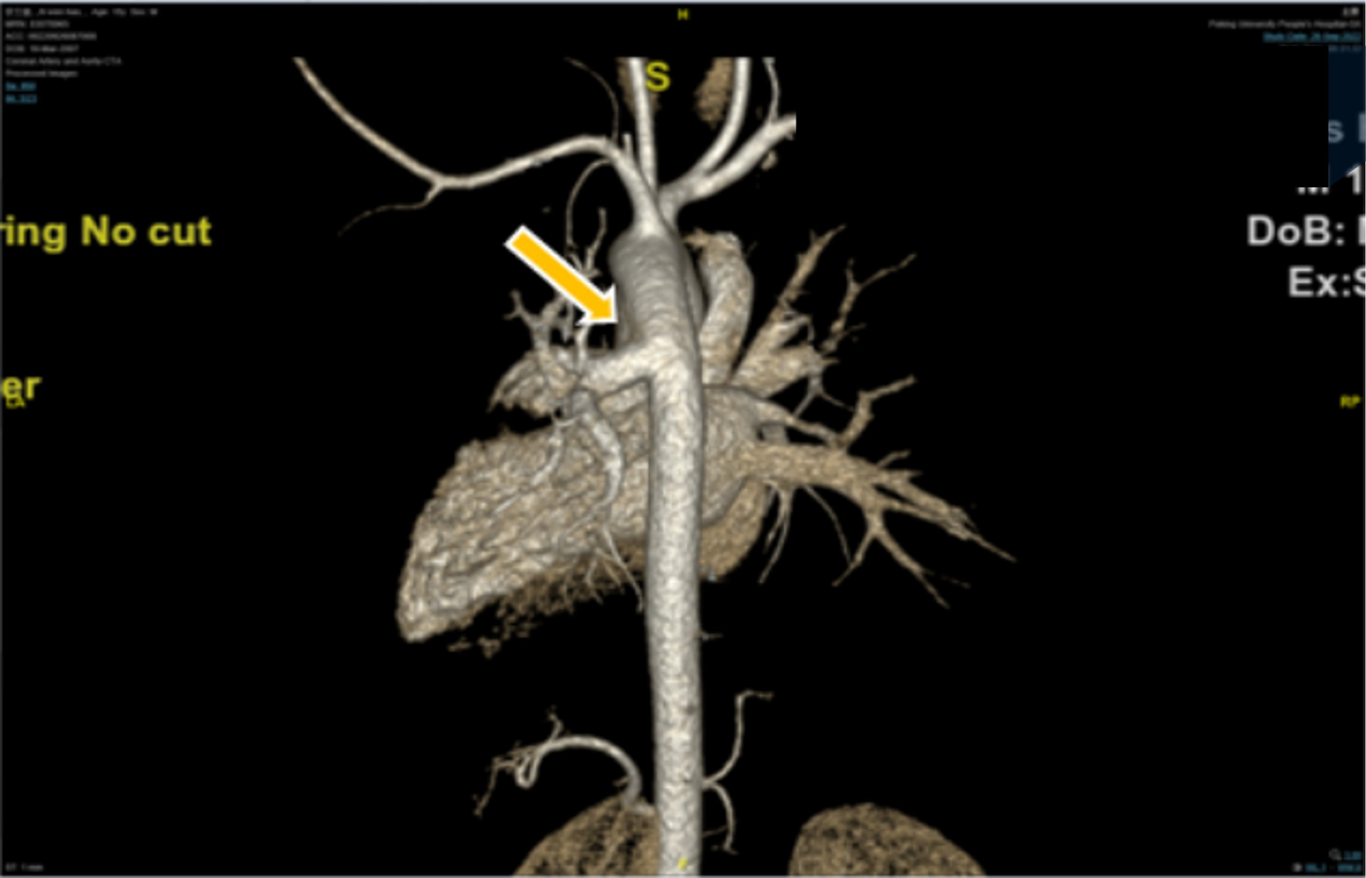
Preoperative three-dimensional angiography. Anomalous origin of the left pulmonary artery from descending aorta is indicated by the yellow arrow.

**Figure 2:**
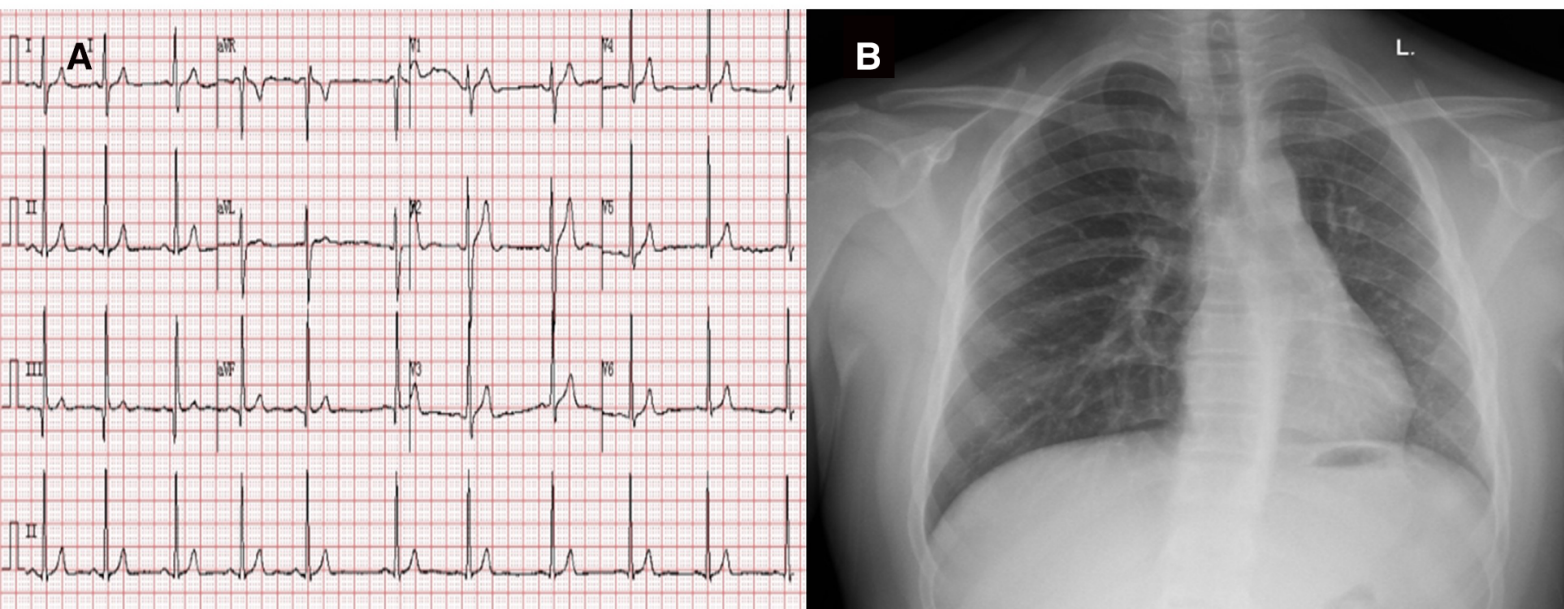
Preoperative ECG (**A**) and chest x-ray (**B**). Preoperative electrocardiogram indicated sinus arrhythmia and chest x-ray showed normal chest image.

A 15-year-old boy (182 cm/90 kg) with a body mass index of 27 kg/m^2^, complaining of aggravated hemoptysis during recent 2 months, was scheduled to undergo selective corrective surgery for AOLPA from the descending aorta (Figure 1). There is no other medical history except for 10 years of stable immunoglobulin A vasculitis (serum creatinine = 47 μmol/L). Preoperative transthoracic echocardiography (TTE) indicated mild tricuspid regurgitation with estimated pulmonary artery systolic pressure of 38 mmHg of the right lung and left ventricular end diastolic dimension of 6.2 cm. Cardiac catheterization before surgery indicated that the pulmonary artery pressure (PAP) of the left lung was same as the systemic artery pressure. ECG and chest x-ray before the operation were normal (Figure 2). The remaining preoperative physical, imaging, and laboratory examinations were normal. Intubation was not difficult after airway evaluation by an anesthesiologist, and the patient was graded as American Society of Anesthesiologists grade III.

Supplemental oxygen was administrated at 5 L/min *via* a mask in the operating room. Noninvasive blood pressure, five-lead ECG, and pulse oxygen saturation were monitored with the baseline value at 120/50 mmHg, 97 bpm, and 100%, respectively. General anesthesia induction began after arterial and peripheral venous cannulation were available. The induction drugs included midazolam (0.05 mg/kg), etomidate (0.2 mg/kg), sufentanil (1.5 μg/kg), and cis-atracurium (0.2 mg/kg). Transesophageal echocardiography (TEE) probe and Swan–Ganz catheter were inserted after a double-lumen tube was intubated. The initial PAP was 42/15 mmHg (mean pulmonary artery pressure = 24 mmHg; pulmonary capillary wedge pressure = 15 mmHg) and the cardiac output was 5.6 L/min. Preoperative arterial blood gas (ABG) was as follows: pH 7.40, PaCO_2_ 39.2 mmHg, PaO_2_ 224 mmHg, hemoglobin 13.0 g/dL, hematocrit 39%, K^+^ 3.87 mmol/L, Ca^2+^ 1.30 mmol/L, glucose 5.54 mmol/L, lactate 1.5 mmol/L, and standard base excess 0.2 mmol/L (inspired oxygen fractions, FiO_2 _= 30%). There are no hemodynamic fluctuations before incision maintaining around 110/60 mmHg.

The detachment of AOLPA from the descending aorta was performed under one-lung ventilation with a right lateral recumbent position. FiO_2_ was increased to 90% to avoid hypoxemia. Meanwhile, proper positive end expiratory pressure (PEEP) (5 mmHg) and intermittent recruitment maneuvers were considered. Before the left pulmonary artery and descending aorta were clamped, intermittent intravenous nicardipine (0.1 mg) was administrated. Cross-clamp time of the descending aorta and left pulmonary artery were 20 min and 40 min respectively. After reperfusion of descending aorta, intravenous norepinephrine (5 μg) was administered for better peripheral circulation. During the whole process of detachment, mean arterial pressure (MAP) was maintained at the level of 60 mmHg.

Subsequently, the process of anastomosing AOLPA to the main pulmonary was performed with the aid of cardiopulmonary bypass (CPB) under mild systemic hypothermia. During CPB, MAP was maintained above 50 mmHg. Hypotension was treated by fluid therapy including blood from the cell saver. After AOLPA was anastomosed to the main pulmonary artery trunk, MAP was maintained at a level of 80 mmHg *via* norepinephrine (0.04–0.06 μg/kg/min) and dopamine (3 μg/kg/min). The patient's temperature was maintained at approximately 36°C all the time. Tranexamic acid was continuous administrated at the rate of 800 mg/h after the bolus of 1,000 mg. Autologous blood transfusion was applied initially, and the patient did not receive allogeneic red blood cells. The surgery lasted for 470 min with fluid positive balance at 400 ml (intake volume 3,600 ml, blood loss 2,000 ml, urine volume 1,200 ml).

## Follow-up

**Figure 3:**
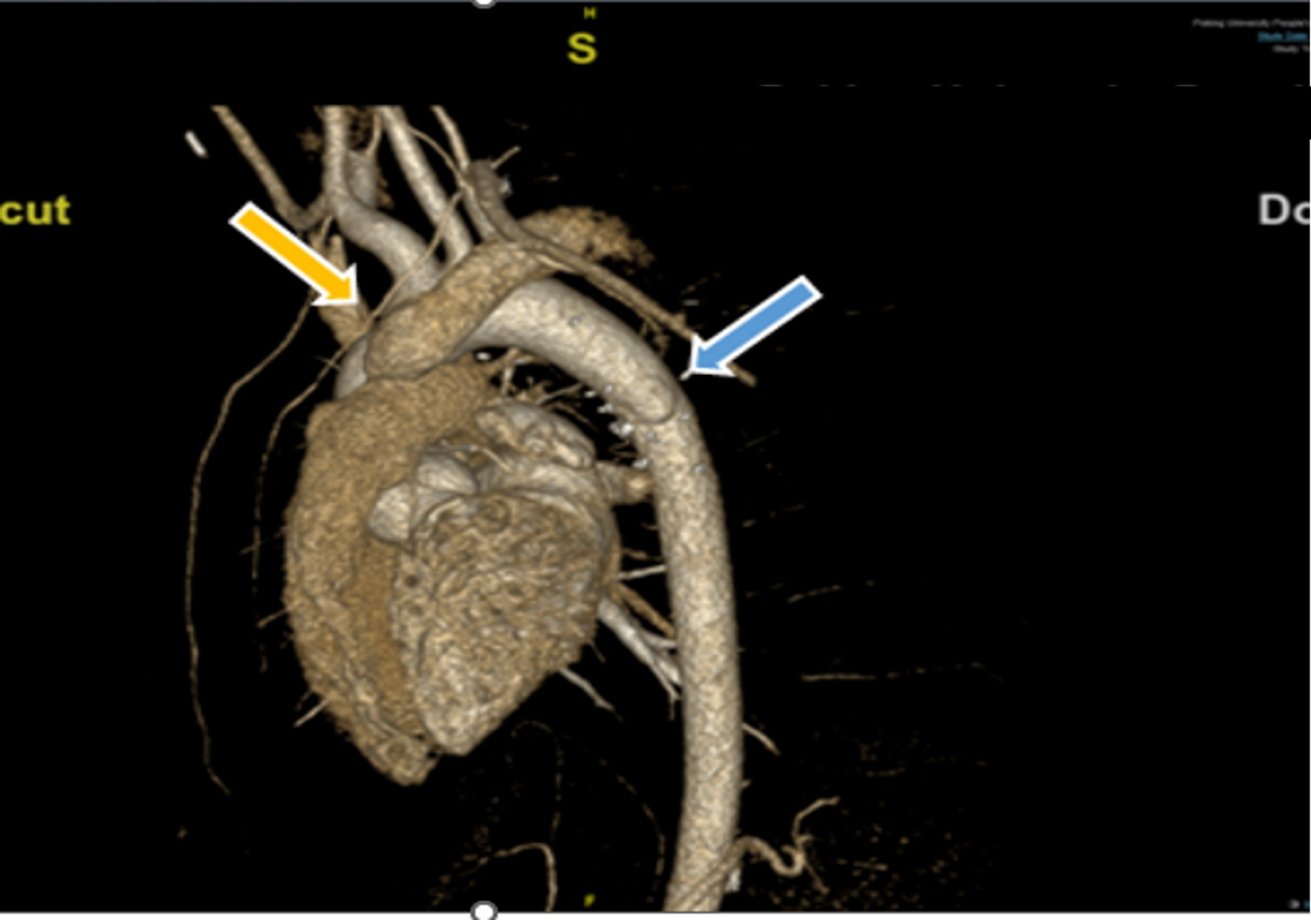
Postoperative volume-rendered cardiac CT angiography image. Repaired descending aorta with pericardium patch was showed as the blue arrow. AOLPA anastomosed to the main pulmonary artery *via* 18# synthetic vessel is indicated by the yellow arrow. AOLPA, anomalous origin of the left pulmonary artery; CT, computed tomography.

The patient survived the surgery with stable hemodynamics (110/58 mmHg, 98 bpm, 100%) and extubated 5 h after the surgery in the intensive care unit (ICU). He received patient controlled intravenous analgesia with a standard regimen of sufentanil (basal infusion at 2 mg/h, 3 mg bolus, and 15-min lockout intervals). Twenty-one hours later, he was transferred from ICU to the ward of Department of Cardiac Surgery. Warfarin was administrated to prevent the thrombosis after surgery, and the dosage was adjusted according to international normalized ratio (INR) constantly. Postoperative volume-rendered cardiac computed tomography (CT) angiography image indicated satisfying correction results (Figure 3). Postoperative TTE estimated pulmonary artery systolic pressure of 29 mmHg without regional wall motion abnormality. The patient was discharged 27 days after surgery under thorough medical care without postoperative complications.

## Discussion

Anomalous origin of a branch pulmonary artery from the aorta is a rare congenital malformation, which may occur in isolation or associated with cardiac defects such as Tetralogy of Fallot and patent ductus arteriosus (6). The definite pathogenesis about AOLPA is unknown and usually thought to be related to abnormal embryogenesis. Surgical corrective techniques for AOLPA include direct implantation or implantation *via* a synthetic graft or autologous pericardial patch with or without the aid of CPB, and stenosis is the major reason for reoperation (6, 7). The key of treating this malformation is early repair to avoid pulmonary arterial hypertension (PAH) (8). In addition, the left lung exposed to systemic pressure would develop irreversible vascular changes especially pulmonary obstructive disease (9). PAH and irreversible pulmonary vascular changes gradually make anesthesia with significant morbidity and mortality (10). Moreover, pediatric PAH has unique features different from adults, including parenchymal and vascular abnormalities in lung development (11). Multidisciplinary planning, sufficient preoperative assessment, rigorous monitoring tools, and careful intraoperative anesthesia management are paramount for handling this unique and challenging surgery successfully.

There is no doubt that induction of general anesthesia requires adequate opioid to avoid the stimulation of intubation especially using a double-lumen tube. The principle of airway management is a lung protection strategy, especially during one-lung ventilation. We increased FiO_2_ and adjusted mechanical ventilation parameters to avoid hypoxemia and hypercarbia, which would further aggravate PAH. PEEP and recruitment maneuvers would be applied to avoid atelectasis, meanwhile paying attention to their influence on PAP.

Among pivotal steps during the surgery, MAP should be adjusted to match the different steps of the procedure. Before AOLPA was detached from the descending aorta, MAP should be maintained at a relatively low but safe level to reduce blood loss. During CPB, hypotension was delt with by fluid volume, sufficient cardiac output and vascular resistance aiming to maintain the stability of PAP. The use of vasopressors and inotropic agents as well as its specific subtypes needed prudent considerations. After AOLPA was anastomosed to the main pulmonary artery trunk, MAP should be maintained at a relatively high level to guarantee the perfusion of left pulmonary artery.

During the whole procedure, blood management is another focus. Surgical correction for AOLPA needed effective hemostatic therapy especially after CPB. Tranexamic acid, factor VIIa, fibrinogen concentrates, and prothrombin complex can be applied if needed. Active temperature management has a positive effect on coagulation and blood loss (12). Cardiovascular surgeries and CPB are associated with intense inflammatory response, the use of ulinastatin and methylprednisolone would produce an anti-inflammatory efficacy (Table 2). However, the patient complained of moderate to severe pain at the initial days after surgery, and a polypill consisting oxycodone and acetaminophen was given as rescue analgesia, indicating that a multimodal analgesia including peripheral nerve block is warranted in the future. Moreover, general anesthesia combined with regional techniques may provide a better analgesia and reduce stress response for early recovery at the same time (13).

**Table 2:** A summary on the perioperative anesthesia management for AOLPA.

Critical steps during the procedure	Caveats	Appropriate anesthesia strategies
Induction of general anesthesia	PAH	Adequate opioid
One-lung ventilation	PAH	Lung protection strategy, avoid hypoxemia and hypercarbia, PEEP, recruitment maneuvers
Detachment of AOLPA	Blood loss	Relatively low MAP with vasodilators
CPB	Hypotension	Fluid volume, sufficient cardiac output
	Coagulation disorder	Tranexamic acid, factor VIIa, temperature management
	Stress response	Ulinastatin, methylprednisolone
Anastomosis of AOLPA	Insufficient perfusion	Relatively high MAP with vasoconstrictors

AOLPA, anomalous origin of the left pulmonary artery; CPB, cardiopulmonary bypass; MAP, mean artery pressure; PAH, pulmonary arterial hypertension; PEEP, positive end expiratory pressure.

Finally, a little pitfall should be emphasized. During the operation, the patient was initially in a lateral position for the manipulation of AOLPA originating from the descending aorta. Then, the patient was transferred to a supine position for anastomosing AOLPA to the main pulmonary. The airway pressure was suddenly raised with MAP changing from 72 to 46 mmHg at the same time. A possibility of pneumothorax was realized, because the surgeon did not insert a chest tube at the end of manipulation on AOLPA in the lateral position.

## Conclusion

In summary, a case with AOLPA from the descending aorta is rare but important, a thorough preoperative evaluation and scrupulous perioperative anesthetic management would obtain an excellent outcome.

## Data Availability

The raw data supporting the conclusions of this article will be made available by the authors, without undue reservation.

## References

[1] Gnanappa GK Laohachai K Orr Y Ayer J. Isolated anomalous origin of left pulmonary artery from the descending aorta: an embryologic ambiguity. Ann Thorac Surg. (2016) 102:e439–41. 10.1016/j.athoracsur.2016.04.089 27772603

[2] Kutsche LM Van Mierop LH. Anomalous origin of a pulmonary artery from the ascending aorta: associated anomalies and pathogenesis. Am J Cardiol. (1988) 61:850–6. 10.1016/0002-9149(88)91078-8 3354450

[3] Prifti E Bonacchi M Murzi B Crucean A Bernabei M Luisi VS Anomalous origin of the left pulmonary artery from the aorta. Our experience and literature review. Heart Vessels. (2003) 18:79–84. 10.1007/s10380-002-0684-7 12756604

[4] Peng Y Hansong S Jun Y. Off cardiopulmonary bypass for patients with anomalous origin of single pulmonary artery branch. Chin J Min Inv Surg. (2013) 13:651–52, 666.

[5] Rajanbabu BB Chigullapally R. Anomalous left pulmonary artery: rare case, uncertain embryology and off-pump approach. Indian J Thorac Cardiovasc Surg. (2019) 35:226–9. 10.1007/s12055-018-0770-8 33061012 PMC7525638

[6] Garg P Talwar S Kothari SS Saxena A Juneja R Choudhary SK The anomalous origin of the branch pulmonary artery from the ascending aorta. Interact Cardiovasc Thorac Surg. (2012) 15:86–92. 10.1093/icvts/ivs110 22467006 PMC3380986

[7] Abu-Sulaiman RM Hashmi A McCrindle BW Williams WG Freedom RM. Anomalous origin of one pulmonary artery from the ascending aorta: 36 years’ experience from one centre. Cardiol Young. (1998) 8:449–54. 10.1017/S1047951100007101 9855098

[8] Salaymeh KJ Kimball TR Manning PB. Anomalous pulmonary artery from the aorta via a patent ductus arteriosus: repair in a premature infant. Ann Thorac Surg. (2000) 69:1259–61. 10.1016/S0003-4975(99)01428-9 10800835

[9] Corno AF Tozzi P Genton CY von Segesser LK. Surgically induced unilateral pulmonary hypertension: time-related analysis of a new experimental model. Eur J Cardiothorac Surg. (2003) 23:513–7. 10.1016/S1010-7940(03)00025-3 12694769

[10] McGlothlin D Ivascu N Heerdt PM. Anesthesia and pulmonary hypertension. Prog Cardiovasc Dis. (2012) 55:199–217. 10.1016/j.pcad.2012.08.002 23009916

[11] Galiè N Humbert M Vachiery J-L Gibbs S Lang I Torbicki A 2015 ESC/ERS guidelines for the diagnosis and treatment of pulmonary hypertension: the Joint Task Force for the Diagnosis and Treatment of Pulmonary Hypertension of the European society of cardiology (ESC) and the European respiratory society (ERS): endorsed by: Association for European Paediatric and Congenital cardiology (AEPC), International Society for Heart and Lung Transplantation (ISHLT). Eur Heart J. (2016) 37:67–119. 10.1093/eurheartj/ehv317. 26320113

[12] Rauch S Miller C Bräuer A Wallner B Bock M Paal P. Perioperative hypothermia—a narrative review. Int J Environ Res Public Health. (2021) 18:8749. 10.3390/ijerph18168749. PMC8394549 34444504

[13] Iwasaki M Edmondson M Sakamoto A Ma D. Anesthesia, surgical stress, and “long-term” outcomes. Acta Anaesthesiol Taiwan. (2015) 53:99–104. 10.1016/j.aat.2015.07.002 26235899

